# Accelerated Stability and Chemical Kinetics of Ethanol Extracts of Fruit of Piper sarmentosum Using High Performance Liquid Chromatography

**Published:** 2011

**Authors:** Hussain Khalid, Ismail Zhari, Sadikun Amirin, Ibrahim Pazilah

**Affiliations:** a *School of Pharmaceutical Science, Universiti Sains Malaysia, Pulau Pinang-11800, Malaysia.*; b *University College of Pharmacy, University of the Punjab, Lahore-54000, Pakistan.*

**Keywords:** *Piper sarmentosum*, Piperaceae, Stability, Pellitorine, Sarmentine, Sarmentosine

## Abstract

The extracts of *Piper sarmentosum, *a medicinal plant, are being used to prepare phytopharmaceuticals while the information about chemical kinetics of constituents of the extract is unavailable to assign precise shelf life (t_90_) and find optimum storage conditions of the product for patient safety, and to avoid economic repercussions of launching an unstable product.

The extract was exposed to three different conditions of high temperature and relative humidity (RH) for six months. The samples were then analyzed at 0, 1, 2, 4 and 6 months by high performance liquid chromatography (HPLC) using pellitorine, sarmentine and sarmentosine as markers. Different chemical kinetic parameters of the markers were evaluated by Arrhenius equation to predict shelf life (t_90_) at different storage conditions and at room temperature.

The markers in the extract followed the zero order degradation, and the activation energy, pre exponential factor and rate constant of the reaction of the markers were found to be varying in samples stored at different conditions. The contents of the markers were found to be decreasing at high temperature and humidity with the passage of time. The predicted shelf life (t_90_) of the markers at room temperature was found to be 16 months approximately.

Results of this study indicate that extracts of the plant are stable at room temperature for 16 months. Moreover, the chemical kinetic data of the markers and the analytical method used to quantify the markers may be useful for phytopharmaceutical industry to produce efficacious and stable products from extracts of the plant.

## Introduction

*Piper sarmentosum *Roxb. (Family: *Piperaceae*), a plant of tropical and subtropical region, is well known due to its culinary and medicinal properties. The extracts of different parts of the plant are being used traditionally to cure various ailments (1-4). The plant has also shown a number of pharmacological activities such as anti-amoebic (5), antibacterial (6), anti-TB (7), antineoplastic (3), neuromuscular blocking (8), hypoglycemic (9), antimalarial (10), antioxidant (11-13) and antiangiogenic (14). Based on these well documented activities, natural product industry has started producing different phytopharmaceuticals from extracts of the plant. Despite the increase in demand of natural product, it is difficult for these products to get in the main stream of pharmaceuticals due to inconsistency in quality, efficacy and safety. The stability testing is important to ensure the standard dose-delivery of a drug throughout its shelf life and to fulfill legal requirements concerning identity, purity, strength and quality of a drug. Furthermore, it helps to avoid economic repercussions of developing and marketing an unstable product. Hence, stability testing of herbal products like any other pharmaceutical product is important to make herbal remedies, reliable medicines.

Unlike pharmaceuticals, the literature regarding stability of herbal remedies is scant, which may be due to inadequate analytical methods and standards or scarce reporting. Since the quantification of constituents in herbal products is difficult, assumptions are often made in a way that the purity and potency is retained as long as appearance of a product remains unchanged, which is un-scientific. Therefore, it is imperative to carry out proper stability studies on extracts as well as herbal products.

A number of methods such as metabolomic fingerprint profiling and quantification of marker compounds of different categories are being used for stability assessment of herbal products, which have both merits and demerits (15, 16). The former is more informative, but the data obtained cannot be used for precise calculations while the later helps to quantify the markers of various categories for the determination of different chemical kinetic parameters to be used for predicting shelf life (t_90_) and optimum storage conditions. Recently, Hussain *et al. *(2009) have investigated the extracts of *Piper sarmentosum *for stability using metabolomic fingerprint profiles obtained through Fourier Transform Infrared spectroscopy and high performance thin layer chromatography (17). The markers are characteristic compounds to a particular plant which signify the presence of active constituents in the extract/herbal product. Keeping it in view, we have used three bioactive markers, pellitorine, sarmentine and sarmentosine, for the determination of chemical kinetic parameters and to evaluate accelerated stability. The study has been designed as per guidelines published by the International Conference on Harmonization (18). 

Based on literature review, the extracts of the plant have not been investigated for accelerated stability and chemical kinetics by applying analytical markers. Therefore, the present study has been undertaken to perform accelerated stability studies on extracts of *Piper sarmentosum*, which may be helpful for natural pharmaceutical industry in preparing and marketing stable products. The novelty of the present work is that the extracts of an important medicinal plant have been investigated scientifically for the first time using a modern technique to predict shelf life (t_90_) in a short time and to investigate chemical kinetics to find optimum storage conditions. The analytical method used for the quantification of markers may be useful for standardization of products made from this plant. Moreover, the results of the study may be a source of information to establish stability of natural products scientifically rather than mere assumptions.

## Experimental

*Plant material, extraction and chemicals*

The fruit of the plant was collected from the Botanical Garden of the School of Pharmaceutical Sciences, Universiti Sains Malaysia and authenticated by Prof. Dr. Zhari Ismail, Herbal Secretariat, School of Pharmaceutical Sciences, Universiti Sains Malaysia, where a voucher specimen was deposited vide reference No. 0071/06. The fruit was cleaned, sliced into small pieces, dried at 40°C and pulverized. The pulverized fruit material (50 g) was extracted twice with 300 mL ethanol by reflux. The extract was filtered and dried in vacuo at 40°C.

The chemicals and solvents of analytical or HPLC grade procured from Merck included ethanol, methanol and acetonitrile. In-house purified HPLC grade water was used while pellitorine, sarmentine and sarmentosine, isolated and characterized previously were used as analytical markers.

*Development and validation of HPLC method*

*Preparation of standard solutions*

The stock solution of pellitorine, sarmentine and sarmentosine were prepared in methanol to a concentration of 50 μg/mL. A series of working standard solutions were prepared by diluting the stock with mobile phase to get solutions of concentration 0.01, 0.10, 0.50, 1.00 and 1.50 μg/mL for pellitorine and sarmentine, while 0.08, 0.80, 4.00, 8.00 and 1.20 μg/mL for sarmentosine.

*Instrumentation*

The samples were analyzed by HPLC system (1100 series, Agilent Technologies, Waldronn, Germany) equipped with degasser (G1379 A), quaternary pump (G1311 A), auto sampler (G1313 A), column oven (G1316 A) and UV detector (G 1314 A).

C*hromatographic conditions*

The samples (15 μL) were eluted by an isocratic mobile phase comprising of methanol: water : acetonitrile (80 : 15 : 5, v/v/v) at flow rate of 1 mL/min. The elution time was 15 min and detection was carried out at 260 nm. Column (Hiber Rt 250-4, LiChrosorb RP 18, 10μm, Agilent Technologies) was maintained at 25°C. The detector was operated in a sensitivity range of 0.005 AUFS with output of 15 mV. The data acquisition was performed by ChemStation version A. 08.03.

*Linearity, limit of detection (LOD), Limit of Quantification (LOQ) and recovery*

Linearity of the method was evaluated by plotting concentration versus peak area of each of the markers over the whole range investigated. Calibration curves of all the standards were constructed by plotting concentration versus peak area in a range of 0.01-1.50 μg/mL for pellitorine and sarmentine, while 0.08-12.00 μg/mL for sarmentosine, and linearity was evaluated by correlation coefficient (R^2^) and standard deviation (SD). The lowest limit of detection (LOD) values of pellitorine, sarmentine and sarmentosine were determined by analyzing the standard solutions successively in two fold dilution with the mobile phase at signal to noise (S/N) ratio 3 : 1 while the lowest limit or quantification (LOQ) was taken at S/N ratio10 : 1. 

Three working standard solutions of pellitorine and sarmentine (0.01, 0.50 and 1.50 μg/mL) and sarmentosine (0.08, 4.00 and 12.00 μg/mL) were used to determine recovery, intraday and inter-day accuracy and precision of the method. For intraday accuracy and precision each standard was analyzed 6 times in same day and quantified at 5 data point calibration while for inter-day accuracy and precision each of the standard solutions was analyzed in triplicate for 6 consecutive days.

For extraction recovery, 200 mg of the fruit powder was spiked separately with standard solutions which were used for accuracy and precision, and extracted with 15 mL ethanol as mentioned in the extraction. The extracts were filtered, dried at 40°C and dissolved in methanol to make solution of a concentration 1 mg/mL. The same quantity of the powder, without spike, was also extracted as a control. The extraction recovery of each of the markers was calculated as a percentage using the following equation:

$\mathrm{\%age recovery}=\frac{100(\mathrm{value obtained}-\mathrm{value of control})}{\mathrm{spiked value}}$

*Stability study protocol*

Study protocol of the International Conference on Harmonization (ICH) as suggested by the Working Party of Herbal Medicinal Products (WPHMP) of the European Agency for the Evaluation of Medicinal Products (18, 19), was applied. The extracts kept in screw caped transparent glass bottles were exposed to three different storage conditions of temperatures and relative humidity such as 30°C/60% RH, 40°C/75% RH and 60°C/85% RH for 6 months. The humidity was controlled by saturated salt solution (20, 21, 22, 23). The samples taken at 0, 1, 2, 4 and 6 months were analyzed in triplicate by HPLC.

*Preparation of sample solutions and analysis*

The stock solution of the extract was prepared in methanol to a concentration of 2 mg/mL while working sample solution (0.2 mg/mL) was prepared by diluting the stock solution with mobile phase. All the working sample solutions were filtered by 0.45 μm polytetrafluoroethylene (PTFE) syringe filter (Whatman, Maidstone, England). All the samples were analyzed in triplicate by HPLC.

*Calculations of chemical kinetic parameters*

*Order of the reaction*

The order of the reaction was determined using the graphic method (24, 25). Zero order, first order and second order graphs were plotted for each temperature. The correlation coefficient of each of the graphs was evaluated and the plot with best linearity was taken as the order of the chemical reaction. The reaction rate constant (K) of the chemical reaction at each elevated temperature was calculated from slope of the curve of % remaining concentration versus time for zero order, natural logarithm of % remaining concentration versus time for first order and inverse of remaining concentration (1/C) versus time for second order.

*Activation energy*

Activation energy (Ea), the energy required to move a molecule from initial state to the transitional state (which is frequently constant) or the fraction of molecules having sufficient energy at a given temperature (A), was determined from the rate constant (K) by plotting logarithm (log K) or natural logarithm (ln K) versus reciprocal of the absolute temperature (1/T) (25). The slope of the straight line of the plot (–Ea /2.303 R or –Ea /R) and intercept (log A or ln A) were used to calculate Ea and A, respectively. The Arrhenius relationship was then used to determine the reaction rate constant at room temperature (25°C, 298.15 K).

The Arrhenius equation is given as follows:

K = A ^e Ea^ ^/ RT^

Ln K = ln A – Ea /RT

Log K = log A – Ea/RT

Where K is a rate constant, A is frequency or collision factor, e is the base of natural logarithm, Ea is activation energy (J mol^-1^), R is the universal gas constant (8.314 J mole^-1^k^-1^) and T is temperature (Kelvin).

*Shelf life (t*_90_ *)*

Shelf life depends on order of the reaction and is calculated using Arrhenius equation. The rate constant at different temperatures was used to estimate shelf life at various temperatures by the following equation for zero order reaction.

Shelf life (t_90_) = 0.105/K

*Statistical analysis*

All the samples and standards were analyzed in triplicate and results were averaged with standard deviation.

## Results and Discussion

**Figure 1:**
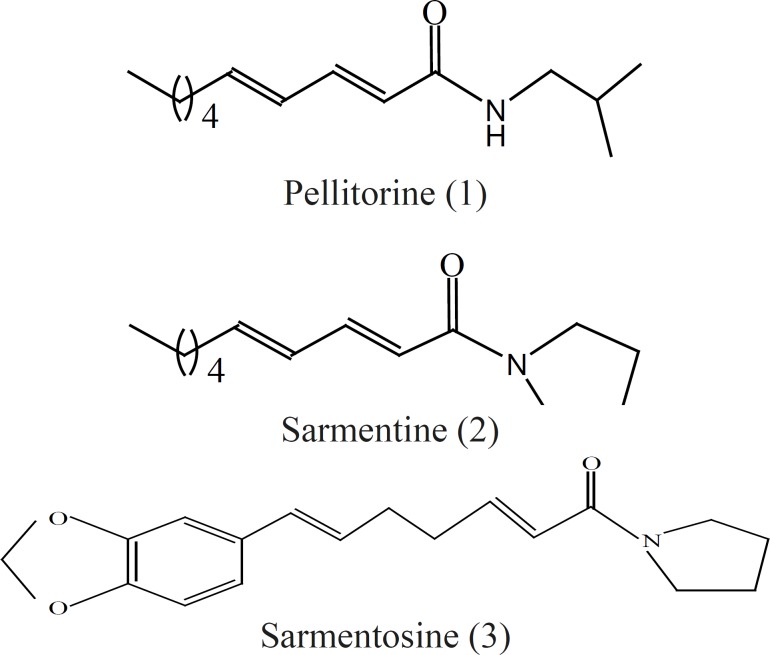
Chemical structures of marker compounds

The chemical structures of three markers, pellitorine, sarmentine and sarmentosine, are given in Figure 1. 

**Figure 2:**
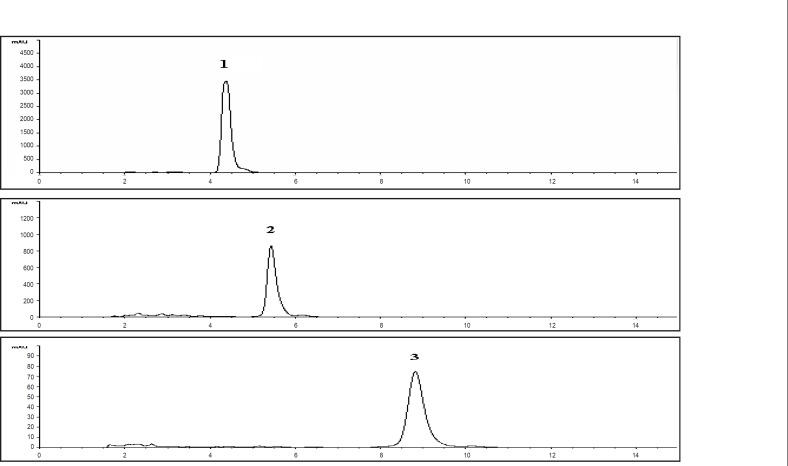
HPLC chromatograms of the markers (pellitorine (1), sarmentine (2) and sarmentosine. (3) at 260 nm

**Figure 3:**
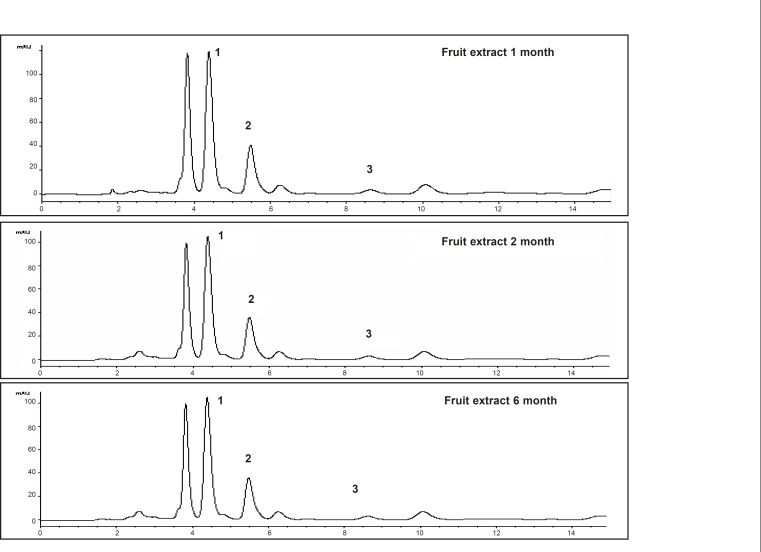
HPLC chromatograms from different samples of ethanol extract for the fruit of *Piper sarmentosum *stored at 30°C/45% RH, detection at 260 nm

These markers were used to develop an analytical method to investigate stability of extracts of *Piper sarmentosum*. The markers showed the maximum absorbance at 260-265 nm. Therefore, the detection was carried out at 260 nm and the optimum resolution of the markers was achieved by eluting the sample with solvent system comprising of methanol: water : acetonitrile (80 : 15 : 5 v/v/v). The chromatograms of the markers and the extracts are given in Figure 2 and 3, respectively, which have shown the separation of the markers in the extract.

The method was found to be linear over the whole range investigation with correlation coefficient 0.999 to 1.000 and standard deviation less than 5%. LOD values of pellitorine, sarmentine and sarmentosine were found to be 0.003, 0.003, and 0.020 μg/mL, respectively, while LOQ values were taken as 0.01, 0.01 and 0.080 μg/mL, respectively. Mean percentage recovery of pellitorine, sarmentine and sarmentosine was found to be 98.52% to 100.00%, 98.83% to 100.13% and 97.77% to 100%, respectively, with relative standard deviation (RSD) less than 5%. Intraday and inter-day analysis accuracy values of the markers were 97.97% to 100.19% with RSD < 5%. These results indicated that the method was reliable, repeatable, reproducible, easy and specific. 

The percentage remaining contents of the markers in the extracts stored at different storage conditions for 6 months have been presented in Table 1. These results indicated that the rate of decomposition was faster at elevated temperature. The loss of the markers was less than 10% in the extracts stored at 30° C/65 % RH indicating the integrity of the extracts with reference to the markers, whereas the decrease in concentration was found to be 25 % and 40 % at 40° C/75 % RH and 60° C/85 % RH, respectively. The results were found to be in accordance with the study mentioning the rise in decomposition with increase in temperature (26). Our results are supported by findings of another study that the rate of a chemical reaction increases by a factor between 2-3 times for each 10°C rising in temperature (25).

**Table 1:** Remaining percentage of pellitorine, sarmentine and sarmentosine in ethanol extracts of fruit of *Piper sarmentosum *stored for 6 months under different storage conditions

**Storage conditions**	**0 Month**	**1 Month**	**2 Month**	**4 Month**	**6 Month**
**% remaining of pellitorine**					
**30°C/60%RH**	**100**	99.99 ± 1.70	98.84 ± 3.46	98.08 ± 2.96	96.77 ± 3.42
**40°C /75%RH**	**100**	98.83 ± 0.65	85.67 ± 0.45	75.65 ± 0.03	73.46 ± 1.34
**60°C /85%RH**	**100**	97.32 ± 2.27	79.80 ± 0.25	74.32 ± 0.74	61.34 ± 0.64
**% remaining of sarmentine **					
**30°C /60%RH**	**100**	99.90 ± 0.22	99.33 ± 0.99	98.36 ± 0.91	96.95 ± 1.32
**40°C /75%RH**	**100**	95.55 ± 1.01	94.27 ± 2.21	92.19 ± 0.57	78.21 ± 1.52
**60°C /85%RH**	**100**	88.65 ± 1.41	76.03 ± 2.51	69.53 ± 0.66	58.89 ± 8.7
**% remaining of sarmentosine **					
**30°C /60%RH**	**100**	98.90 ± 2.23	96.95 ± 0.08	95.33 ± 0.04	94.96 ± 0.12
**40°C /75%RH**	**100**	91.82 ± 0.95	87.73 ± 0.28	86.51 ± 1.15	74.80 ± 0.08
**60°C /85%RH**	**100**	91.11 ± 0.19	83.04 ± 0.14	82.36 ± 0.35	63.32 ± 0.18
**30°C /60%RH**	**100**	99.90 ± 0.22	99.33 ± 0.99	98.36 ± 0.91	96.95 ± 1.32

**Figure 4:**
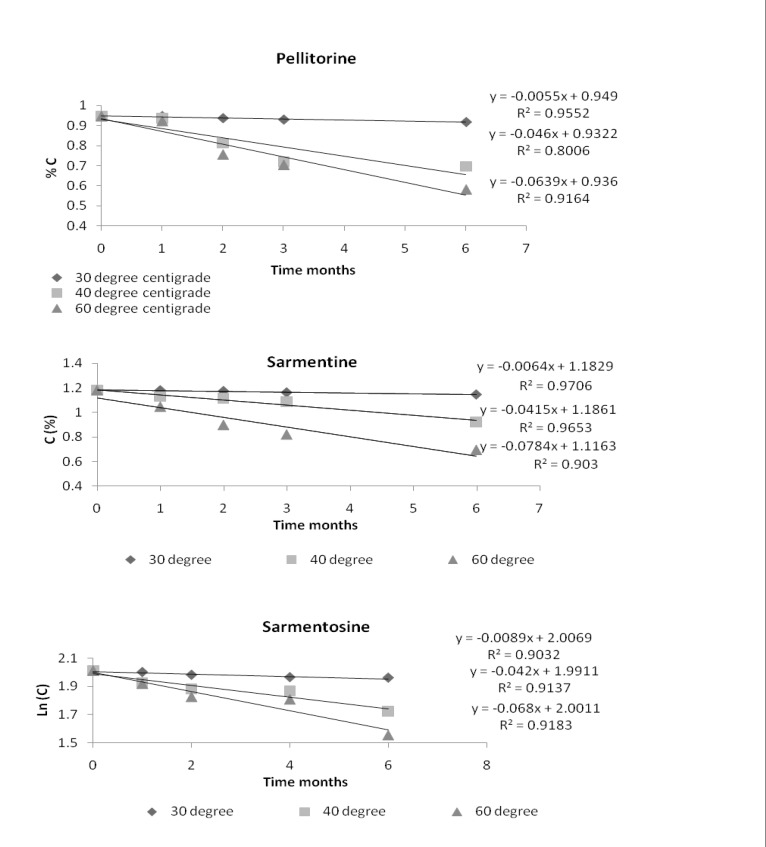
Plot of concentration (%) of the markers in ethanol extract of fruit of *Piper sarmentosum *versus time for zero order reaction

The order of reaction of the markers in the extract was determined at each temperature and the curve with the best linearity was taken as order of the reaction. By comparing different curves, it was found that the markers followed the zero order reaction. The % remaining concentration of the markers versus time graphs showing the zero order reaction are given in Figure 4. 

These results indicated that degradation of the markers was independent on their initial concentration.

**Figure 5:**
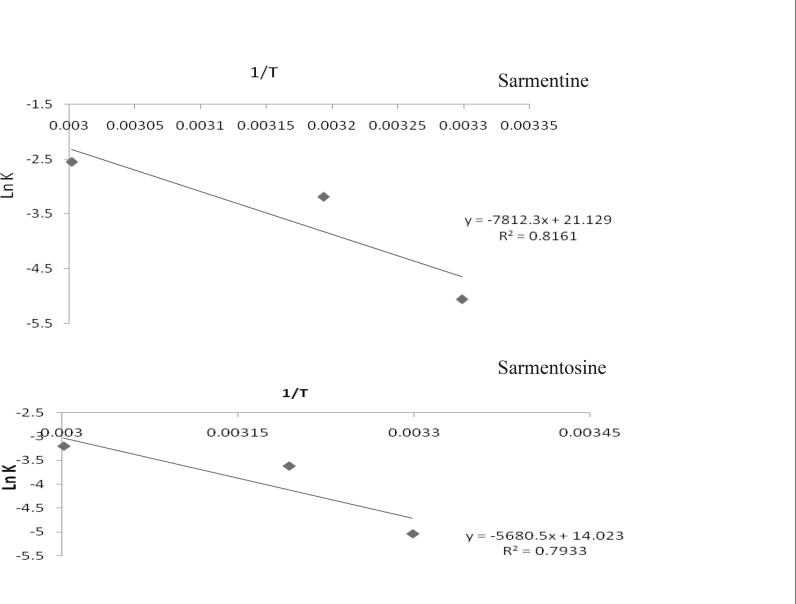
Plot of natural log of rate constant versus inverse of temperature (Kelvin^-1^) of pellitorine, sarmentine and sarmentosine in ethanol extract of fruit of *Piper sarmentosum *at various temperatures, lnK (natural log of rate constant); 1/T (inverse of temperature

The reaction velocity or degradation rate constant (K) of the markers was taken from the slope of their curves of % remaining concentration versus time. Rate constant of each of the markers at room temperature was determined by extrapolating the graph of (ln K) versus inverse of temperature (1/T Kelvin^-1^). The Arrhenius plots of the markers, pellitorine, sarmentine and sarmentosine, are presented in Figure 5 while degradation rate constant (K) of each of the markers at different temperatures is presented in Table 2.

**Table 2:** Rate constant (K), activation energy (Ea) and pre-exponential factor (A) of the markers in ethanol extract of fruit of *Piper sarmentosum *at different temperatures

**Name of marker**	**K** **25°C**	**K** **30°C**	**K** **40°C**	**K** **60°C**	**Ea (KJ mol** ^-1^ **)** **25°C**	**A (S** ^-1^ **)**
**Pellitorine**	0.0062	0.0093	0.021	0.085	67.71	453270665.23
**Sarmentine**	0.0063	0.0096	0.022	0.098	63.39	1500403636.72
**Sarmentosine**	0.0065	.0089	0.0163	0.048	47.24	1230584.73

Ea, a positive product of slope and universal gas constant (R = 8.314 KJ mol^-1^), of each of the markers was determined from slope of the straight line, whereas A was calculated from intercept of the curve. The activation energy and pre-exponential factor of all the markers presented in Table 2 indicated that the activation energy of pellitorine was higher as compared to that of the sarmentine and sarmentosine. Hence, stability of the markers was found in the order as pellitorine > sarmentine > sarmentosine. A similar trend was also found in values of the pre-exponential factor of the markers. 

Since, the markers followed the zero order reaction, t_90_ of each of the markers was determined through dividing 0.105 by degradation rate constant (K). The estimated t_90_ of the markers at different storage conditions is presented in the Table 3.

**Table 3:** Shelf life (t_90_) of the markers in the ethanol extract of fruit of *Piper sarmentosum *at different storage conditions

**Marker**	**t**_90_ **25°C**	**t**_90_ **30°C/60%RH**	**t**_90_ **40°C/75%RH**	**t**_90_ **60°C/85%RH**
**Pellitorine**	16.94	11.29	5	1.24
**Sarmentine**	16.67	10.94	4.77	1.07
**Sarmentosine**	16.15	11.80	6.44	2.17

RH (relative humidity); t_90_ is in months

Being a plant of *Piperaceae*, *Piper sarmentosum *is rich in amide type alkaloids (27 - 29), which have shown different pharmacological activities. Hence, three amides, pellitorine, sarmentine and sarmentosine (characteristics to the plant) were selected as pharmacologically active analytical markers to investigate the extracts of the plant for chemical kinetics. 

Stability studies provide evidences on how the quality of a drug substance varies under the influence of environmental factors over the time (30). Before developing a dosage form, stability studies are the first quantitative assessment of chemical constancy of a product. These studies are also useful to recommend the storage conditions and predict shelf life of medicinal products. The stability testing, which involves examining the quality and potentiality of a product at suitable time intervals is conducted for a period corresponding to the normal time that the product is likely to remain in stock or in use. Degradation is usually slow at room temperature and shelf life may go up to several years. Since the period may be as long as several years, stability testing for such a long period is time consuming and expensive. Hence, stability studies are conducted at high temperatures to predict long term stability in a short time.

Stability of a product is affected by a number of physical factors such as temperature, moisture and light, and chemical factors such as hydrolysis, oxidation, polymerization and isomerisation. Temperature enhances the rate of degradation of active ingredients due to increase in their kinetic energy which results in increasing the fraction of colliding molecules. Moisture contents increase the rate of decomposition and make the product susceptible to hydrolysis (31). In case of herbal crude powders or extracts, it facilitates the growth of microbes which not only deteriorate the constituents but may also produce toxic substances. Decomposition of pharmaceutical preparation due to oxidation is nearly as probable as that with hydrolysis and the rate of oxidation is also temperature dependent. For example, the rate of fatty acids peroxidation accelerates as the temperature exceeds 50°C (32). Polymerizations, addition of similar molecules, isomerisation, and the variation of isomeric forms, are the additional factors affecting the stability. Sunlight as a form of energy also facilitates the degradation and affects the stability of pharmaceuticals (33). The temperature and sunlight are particularly important for extracts containing volatile and photolabile constituents (25, 32). Increase in temperature and exposure to sunlight decreases the activation energy (Ea) and helps molecules to cross the energy barrier to start a reaction.

The marker compounds are amides and do not possess free hydroxyl groups, hence are not accessible to hydrolysis. The two oxygen atoms present on the aromatic ring of sarmentosine are also unavailable for hydrolysis as these are linked with each other through methylene (-CH_2_-) bridge. 

It is concluded from this study that the markers, pellitorine, sarmentine and sarmentosine, followed the zero order degradation reaction. Based on accelerated stability testing of the markers, the extract had shown shelf life (t_90_) of 16 months approximately at room temperature. The decrease in the contents of markers at elevated temperature suggested that raw material and finished product must be stored at room temperature. Moreover, excessive heating during manufacturing process must be monitored and controlled carefully to save the active amides in the formulation.
